# Generalized Lymphadenopathy as the First Manifestation of Systemic Lupus Erythematosus

**DOI:** 10.7759/cureus.30089

**Published:** 2022-10-09

**Authors:** Leonor Soares, André Rebelo Matos, Marta Mello Vieira, Rita Cruz, Umbelina Caixas

**Affiliations:** 1 Internal Medicine Department, Hospital São José, Centro Hospitalar Universitário de Lisboa Central, E.P.E, Lisbon, PRT; 2 Critical Care Medicine Department, Hospital São José, Centro Hospitalar Universitário de Lisboa Central, E.P.E, Lisbon, PRT

**Keywords:** lupus lymphadenitis, constitutional symptoms and sle, lupus lymphadenopathy, lymphadenopathy, sle

## Abstract

Lymphadenopathy (LAP) is a common but nonspecific feature of many diseases, representing a vast spectrum of etiologies such as infectious or inflammatory diseases, malignancies, and drugs. In systemic lupus erythematosus (SLE), it can be the first manifestation. We present the case of a 20-year-old female with a history of fever, night sweats, anorexia, and asthenia for five months. She also had diffuse generalized LAP. Although malignant etiologies were our major concern, an extensive workup for malignancy and infections was unrevealing. However, an autoimmune workup led to the diagnosis of SLE. This case shows that SLE can present as generalized LAP with constitutional symptoms, and hence it should be considered in the differential diagnosis.

## Introduction

Lymphadenopathy (LAP) is characterized by nodes that are abnormal in either number, size, or consistency. According to their distribution, they can be classified as “localized”, if only one region is involved, or “generalized” if they involve two or more non-contiguous regions [1]. It can be associated with a vast spectrum of etiologies such as infectious (viral, bacterial, parasitic, fungal) or inflammatory diseases [connective tissue diseases, Castleman disease, sarcoidosis, Kikuchi-Fujimoto disease (KFD)], malignancies, and drugs [2].

In systemic lupus erythematosus (SLE), LAP represents a benign and common finding. Though it is not considered a clinical criterion in the 2019 European League Against Rheumatism/American College of Rheumatology (EULAR/ACR) classification criteria, some reports have suggested that it can be the first manifestation, with a cumulative incidence ranging from one-third to one-half of the cases [2,3,4,5]. There are some clinical and paraclinical characteristics associated with LAP in SLE, such as more constitutional symptoms (fatigue, fever, and weight loss), more cutaneous symptoms, increased anti-dsDNA antibodies, and decreased complement levels [6,7].

We report the case of a 20-year-old female patient with constitutional symptoms and generalized LAP, in which the first differential diagnosis was a lymphoproliferative disease. However, after an extensive workup for malignant, infectious, and immunologic diseases, a diagnosis of SLE was made.

## Case presentation

The patient was a 20-year-old Caucasian female with a medical history of anxiety disorder, treated with pregabalin 50 mg bid. She also had complaints of asthenia and anorexia with weight loss (8 kg) for five months. Additionally, she had an afternoon fever (maximum axillary temperature of 38.5 ºC), accompanied by night sweats. She was evaluated on multiple occasions in the ED, with normal physical examination and nonspecific analytical alterations (Table 1), besides normocytic and normochromic anemia with hemoglobin of 11.2 g/dL and elevated liver enzymes (aspartate aminotransferase: 63 U/L, alanine aminotransferase: 65 U/L, and lactic dehydrogenase: 316 U/L).

**Table 1:** Initial investigation ALP: alkaline phosphatase; ALT: alanine aminotransferase; AST: aspartate aminotransferase; CRP: C-reactive protein; GGT: gamma-glutamyl transferase; LDH: lactic dehydrogenase; MCH: mean corpuscular hemoglobin; MCV: mean corpuscular volume; PT: prothrombin time; PTT: partial thromboplastin time

Variable	Reference range	Results
Hemoglobin (g/dL)	12.0 – 15.0	11.7
MCV (fL)	78.0 – 96.0	83.0
MCH (pg)	26.0 – 33.0	28.1
White cells count (per uL)	4,500 – 11,000	4,890
Neutrophils (per uL)	2,000 – 8,500	2,760
Lymphocytes (per uL)	900 – 3,500	1,940
Platelets (per uL)	150,000 – 450,000	295,000
PT (sec)	9.4 – 12.5	12.7
APTT (sec)	25.1 – 36.5	28.1
CRP (mg/L)	<5.0	1.9
Urea (mg/dL)	15.0 – 40.0	23
Creatinine (mg/dL)	0.57 – 1.11	0.9
Sodium (mEq/L)	136 – 145	134
Potassium (mEq/L)	3.50 – 5.10	4.4
Chloride (mEq/L)	98 – 107	105
AST (U/L)	5.0 – 34.0	57
ALT (U/L)	0.0 – 55.0	58
GGT (U/L)	9.0 – 36.0	21
ALP (U/L)	40 – 150	52
LDH (U/L)	125 – 220	316
Bilirubin (mg/dL)	0.20 – 1.20	0.44

**Figure 1:**
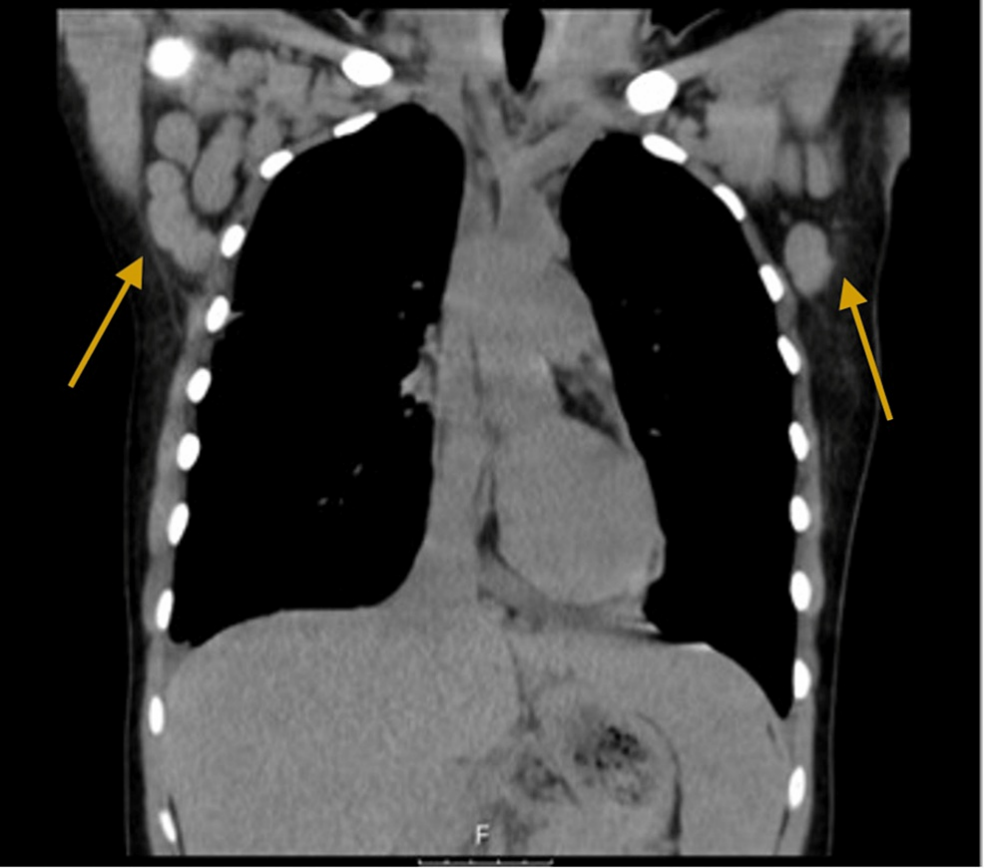
Bilateral axillary enlarged lymph nodes (arrows)

**Figure 2:**
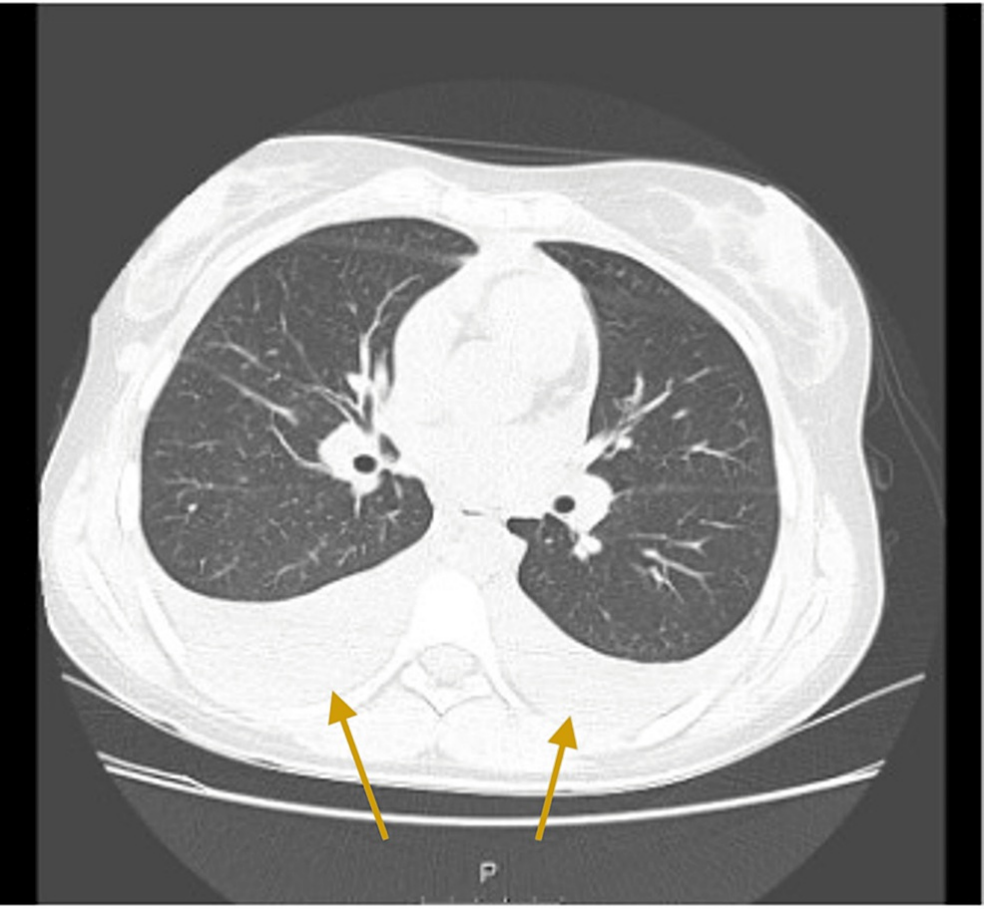
Bilateral pleural effusion (arrows)

The patient was discharged and referred to the Post-Emergency Clinic, where palpable nodules in the right posterior cervical chain and right axilla were found. A chest CT scan was performed, which revealed bilateral axillary, cervical, and mediastinal LAP, and bilateral pleural effusion, with a caesural component on the right and passive atelectasis of the lung parenchyma (Figures 1, 2).

**Figure 3:**
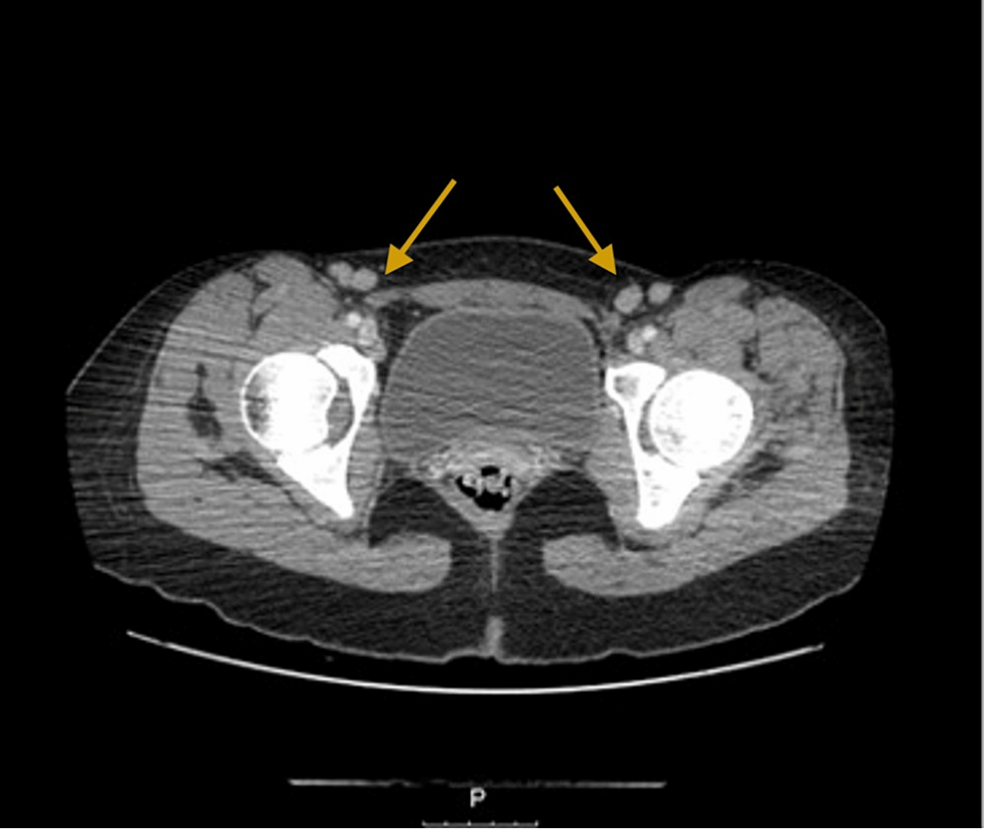
Bilateral inguinal enlarged lymph nodes (arrows)

Faced with a constitutional condition associated with multiple LAP, the patient was admitted to the Internal Medicine ward for further investigations. She underwent an abdominopelvic CT, which revealed LAP at the splenic and hepatic hilum, mesenteric and retroperitoneal (iliac, hypogastric, and inguinofemoral) (Figure 3).

The differential diagnosis included infectious, neoplastic, and autoimmune causes although the suspected diagnosis was lymphoma. Infectious causes were ruled out. Blood and urine cultures were sterile; no mycobacteria were found in the acid-fast bacilli stains, culture, and nucleic acid amplification test from blood and gastric juice; serologies for syphilis, hepatitis A, B and C viruses, HIV, cytomegalovirus (CMV), and Epstein-Barr virus (EBV) were negative.

Autoimmune diseases workup (Table 2) showed positive anti-nuclear antibodies (ANA) (1:640, fine mottled), anti-dsDNA (4,028 IU/ml), and anti-nucleosomes (1,180 U/ml). From the extractable nuclear antigen (ENA) panel, anti-Smith (anti-Sm), anti-ribonucleoprotein (anti-RNP) 70, anti-RNP A, anti-RNP C, and anti-histones were also positive. She also had a high sedimentation rate (53 mm/h) but low C-reactive protein (1.9 mg/L), and low serum complement levels with reduced C3 and C4 fractions (0.71 g/L and 0.09 g/L respectively). Lupus anticoagulant, anti-β2-glycoprotein 1, and anti-cardiolipin tests were negative.

**Table 2:** Autoimmune workup Ab: antibody; ANA: antinuclear antibodies; anti-CCP: anti-cyclic citrullinated peptides; anti-dsDNA: anti-double stranded DNA; anti-DSF: anti-dense-fine-speckled; anti-PM/Scl: anti-polymyositis/systemic sclerosis; anti-PCNA: anti-proliferating cell nuclear antigen; anti-RNP: anti-ribonucleoprotein; anti-Sm: anti-Smith; anti-SSA: anti-Sjögren's syndrome A; anti-SSB: anti-Sjögren's syndrome B; C3: complement 3 protein; C4: complement 4 protein; ENA: extractable nuclear antigen antibodies; ESR: erythrocyte sedimentation rate

Variable	Reference range	Results
ESR (mm/h)	<16	53
ANA		Positive: 1:640
Anti-dsDNA (UI/mL)	Positive: >40	4,028
Anti-nucleosome (U/mL)	Positive: >20	1,180
ENAs		Anti-SSA: negative
		Anti-Ro52KD: negative
		Anti-SSB: negative
		Anti-Sm: positive 2+
		Anti-RNP 70: positive 3+
		Anti-RNP A: positive 3+
		Anti-RNP C: positive 2+
		Anti-Jo-1: negative
		Anti-PM/Scl 100: negative
		Anti-Scl-70: negative
		Anti-centromere: negative
		Anti-PCNA: negative
		Anti-histone: positive 1+
		Anti-ribosomal P: negative
		Anti-PM-Scl: negative
		Anti-nucleosome: positive 1+
		Anti-DSF 70: negative
Anti-CCP (UQ)	Positive: >20	26.8
Rheumatoid factor (UI/mL)	<15	<9.2
C3 (g/L)	0.9 – 1.80	0.71
C4 (g/L)	0.10 – 0.40	0.09

To help exclude a neoplastic cause, an excisional biopsy of the axillary lymph node was performed, which revealed reactive lymphadenitis, without neoplastic tissue, herpesvirus 8, CMV, and EBV. A diagnosis of SLE was made, and the patient was treated with deflazacort (1 mg/kg/day). Her condition improved within two weeks. The fever, asthenia, and enlarged cervical lymph nodes disappeared.

## Discussion

SLE is a chronic autoimmune disease that can virtually affect any organ. It is associated with a multiplicity of clinical and laboratory findings, which makes SLE a diagnostic challenge. Our patient had a fever, anorexia and weight loss, and generalized LAP, but none of the most typical features of SLE. Hence, the diagnosis of SLE was established by ruling out other causes of LAP.

Malignancy was a major concern in our patient, especially given her constitutional symptoms. She underwent a full body CT, which revealed generalized LAP in the axilla, mediastinum, and neck, as well as in the retroperitoneum, hepatic and splenic hilum, and mesenterium. The initial hypothesis was a lymphoproliferative disease. Hodgkin or non-Hodgkin lymphoma may present with painless, firm, peripheral LAP [1]. It is often associated with B symptoms such as fever, night sweats, and weight loss, as in our patient.

Castleman disease is a heterogeneous group of lymphoproliferative disorders. It can be unicentric if it only affects a single region of the body, or multicentric if it involves multiple regions. The multicentric Castleman disease is often associated with systemic symptoms such as fever, generalized LAP, hepatosplenomegaly, and cytopenias. In 50% of the cases, the multicentric Castleman disease is associated with human herpes virus 8 (HHV-8) [8,9].

The patient's laboratory tests showed only mild anemia, with normal leucocyte and platelet count. Lymph node biopsy showed reactive lymphadenitis without neoplastic cells but did not show the classic SLE finding of hematoxylin bodies. And though it makes lymphoproliferative diseases less likely, it did not provide us with a final diagnosis. Infectious causes such as HIV, CMV, EBV, tuberculosis, or syphilis were also considered. However, both serologies and cultures were negative.

The diagnosis was ultimately made with the autoimmune disease panel. The patient presented positive high titers of ANA, anti-dsDNA, anti-SM, and low complement levels. According to EULAR/ACR classification criteria, a score of 10 or more is diagnostic of SLE [10,11]. The laboratory findings along with fever and pleural effusion gave a score of 17 as per the classification criteria. And though it is a common feature, LAP is not a classification criterion. Lupus lymphadenopathy (LL) is typically generalized and can be the first sign of the disease. Some researchers believe that there is a relationship between LL and disease activity, with more constitutional symptoms and a higher need for steroids [6].

Lymph node enlargement in people with previously diagnosed SLE can be due to a flare of the disease (lupus lymphadenitis), infections, KFD, or lymphoproliferative disease. Infections and both Hodgkin and non-Hodgkin lymphomas are more common in SLE patients than in the general population due to immune deficits and dysregulation [12]. There are no specific tests to distinguish between a lymphoid malignancy and reactive LAP due to infection or SLE disease flare. However, lupus lymphadenitis responds quickly to glucocorticoids, decreasing in size.

KFD or histiocytic necrotizing lymphadenitis shares some major features with SLE, such as female preponderance, young age of onset, and clinical presentation with fever, arthralgia, skin eruptions, and lymphadenopathy. The latter characteristics are usually self-limiting and localized, more commonly cervical. A lymph node biopsy is mandatory for the diagnosis as the histopathological characteristics are distinctive to allow its recognition as a specific entity [13]. KFD has been rarely described in association with SLE, in which case it can be a diagnostic challenge.

## Conclusions

Generalized LAP is a common presentation of a wide range of diseases. The diagnostic accuracy depends on medical history, physical examination, selected laboratory tests, and lymph node biopsy. LAP is not a classification criterion in SLE, but it can precede the most typical features of the disease. Lupus lymphadenitis can be the first sign of a flare, but infection, lymphoma, or KFD must be considered in all patients with SLE and LAP.

Due to the nonspecific presentations and multisystem involvement of SLE, it is important to have a high index of suspicion as early diagnosis and treatment can improve survival and quality of life.
